# Endocrine and Local IGF-I in the Bony Fish Immune System

**DOI:** 10.3390/biology5010009

**Published:** 2016-01-26

**Authors:** Anne-Constance Franz, Oliver Faass, Bernd Köllner, Natallia Shved, Karl Link, Ayako Casanova, Michael Wenger, Helena D’Cotta, Jean-François Baroiller, Oliver Ullrich, Manfred Reinecke, Elisabeth Eppler

**Affiliations:** 1 Institute of Anatomy, University of Zurich, Winterthurerstrasse 190, Zurich 8057, Switzerland; anne-constance.franz@gmx.ch (A.-C.F.); oliverfaass@gmx.de (O.F.); nshved@yahoo.com (N.S.); karl.link@iem.uzh.ch (K.L.); mixwenger@hotmail.com (M.W.): oliver.ullrich@anatom.uzh.ch (O.U.); manfred.reinecke@uzh.ch (M.R.); 2 Study Group “Magdeburger Arbeitsgemeinschaft für Forschung unter Raumfahrt- und Schwerelosigkeitsbedingungen” (MARS), Otto-von Guericke-University Magdeburg, Universitätsplatz 2, Magdeburg 39106, Germany; 3 Friedrich-Loeffler-Institute, Federal Research Institute for Animal Health, Greifswald-Insel Riems 17493, Germany; Bernd.Koellner@fli.bund.de; 4 Institute of Evolutionary Medicine, University of Zurich, Winterthurerstrasse 190, Zurich 8057, Switzerland; 5 Institute for Fish and Wildlife Health, University of Bern, Länggassstrasse 122, Bern 3012, Switzerland; ayako.casanova@vetsuisse.unibe.ch; 6 Fish Physiology and Genomics, UMR ISEM—Institut des Sciences de l’Evolution de Montpellier, Département Conservation et Domestication, CIRAD, Campus International de Baillarguet, TA B-116/A, Montpellier cedex 5, Montpellier 34398, France; dcotta@cirad.fr (H.D.C.); baroiller@cirad.fr (J.-F.B.); 7 Institute of Anatomy, University of Basel, Pestalozzistrasse 20, Basel 4056, Switzerland; 8 Institute of Anatomy II, University of Erlangen-Nürnberg, Universitätsstraße 19, Erlangen 91054, Germany

**Keywords:** GH, TNF-α, IGF, receptor, head kidney, spleen, lymphocytes, phagocytes, innate immune system, adaptive immune system

## Abstract

A role for GH and IGF-I in the modulation of the immune system has been under discussion for decades. Generally, GH is considered a stimulator of innate immune parameters in mammals and teleost fish. The stimulatory effects in humans as well as in bony fish often appear to be correlated with elevated endocrine IGF-I (liver-derived), which has also been shown to be suppressed during infection in some studies. Nevertheless, data are still fragmentary. Some studies point to an important role of GH and IGF-I particularly during immune organ development and constitution. Even less is known about the potential relevance of local (autocrine/paracrine) IGF-I within adult and developing immune organs, and the distinct localization of IGF-I in immune cells and tissues of mammals and fish has not been systematically defined. Thus far, IGF-I has been localized in different mammalian immune cell types, particularly macrophages and granulocytes, and in supporting cells, but not in T-lymphocytes. In the present study, we detected IGF-I in phagocytic cells isolated from rainbow trout head kidney and, in contrast to some findings in mammals, in T-cells of a channel catfish cell line. Thus, although numerous analogies among mammals and teleosts exist not only for the GH/IGF-system, but also for the immune system, there are differences that should be further investigated. For instance, it is unclear whether the primarily reported role of GH/IGF-I in the innate immune response is due to the lack of studies focusing on the adaptive immune system, or whether it truly preferentially concerns innate immune parameters. Infectious challenges in combination with GH/IGF-I manipulations are another important topic that has not been sufficiently addressed to date, particularly with respect to developmental and environmental influences on fish growth and health.

## 1. Introduction: The GH/IGF-System and the Immune System

In mammals and in bony fish, a role for the growth hormone (GH)/insulin-like growth factor (IGF)-system in the modulation of the immune system has been under debate for decades. In general, GH is considered a stimulator of the immune system in mammals [1,2,3,4] as well as in bony fish [5,6]. Generally, IGF-I is a potent growth factor that stimulates immune cell growth and differentiation and inhibits apoptosis [1,3].

In mammals and fish, (endocrine) IGF-I is mainly produced in the liver, with GH being the major stimulus for its synthesis and release into the circulation. There is general agreement that the serum IGF-I concentration influences GH release from the anterior pituitary via a negative feedback mechanism by specific inhibition of GH gene transcription and secretion in mammals [7,8,9] and teleosts [10,11,12,13,14]. While the GH/IGF-I axis is generally considered well-preserved throughout phylogeny [13,14,15,16], the role of IGF-II is still enigmatic in fish [11,15]. Furthermore, in bony fish a few years ago, a third IGF (IGF-3) has been postulated to be expressed exclusively in gonads [17]. In tilapia, IGF-3 expression was also detected by real-time PCR in other tissues including head kidney and spleen, but to a much lower extent than in the gonads [18]. The GH/IGF-system of bony fish further consists of multiple subtypes of GH receptors (GHR), IGF receptors and IGF binding proteins that arose through gene duplication events, which occurred during evolution of the teleost lineage (for further reading, see [19]).

In this review, we will mainly focus on the GH/IGF-I axis, particularly since most published data in the context of the fish (and mammalian) immune system concern the axis GH-GHR-IGF-I with some comparative aspects on mammals at the end of the respective chapters.

## 2. GH and IGF-I Actions in Bony Fish Immune Responses

Indications exist that IGF-I plays important roles in cell proliferation, differentiation and functional modulation of the immune system of fish [5,6,20,21]. Studies so far have shown that in the piscine immune system, exogenous administration of GH enhanced innate immune activities of leukocytes [22] and phagocytes [23] and activated rainbow trout and gilthead sea bream phagocytes *in vitro* [24] and *in vivo* [25,26]. Furthermore, GH stimulated the respiratory burst of leukocytes in vibriosis-furunculosis-vaccinated rainbow trout [27] and the plasma lysozyme activity, which is a marker for the innate immune system, in rainbow trout and channel catfish [20,28]. Further, both GH and IGF-I stimulated the proliferation of gilthead sea bream head kidney leukocytes *in vitro* [21]. Thus far in two different fish species, GH treatment was combined with bacterial infection: in rainbow trout challenged with *Vibrio anguillarum*, the bactericidal activity was not enhanced by GH [26]. In another study in channel catfish infected with *Edwardsiella ictaluri*, the mortality was not positively influenced [28]. Nevertheless, the immune systems of these species investigated so far, strongly differ from each other, not only due to their different environments, which is cold water for rainbow trout and warm water for channel catfish. Thus, more studies considering different life traits, environments and pathogens are needed to gain further insights why positive influences of GH on different immune functions may not lead to an efficient immune response and an improved health status.

Innate immune functions are stimulated also by IGF-I: *in vitro* administration of salmon IGF-I, human IGF-I, and IGF-II increased superoxide production in zymosan-stimulated rainbow trout head kidney leukocytes, and salmon IGF-I had a significant effect on superoxide production, which was observed to be equipotent to that of salmon GH [29]. Two IGF type-I receptors (IGFR Ia and IGFR Ib) and GHR1 and GHR2 genes were detected in brain, pituitary, liver, and gills, as well as in head kidney leukocytes, except IGFR Ia gene expression was absent in head kidney leukocytes. In the same study, *in vivo* intraperitoneal injection of salmon IGF-I increased the plasma levels of lysozymes [29]. In summary, these data mainly point to the stimulation of innate immune reactions including cell expansion, particularly of phagocytes by both, GH and IGF-I.

Although, admittedly, the majority of published reports examined only innate immune parameters and did not consider adaptive immune parameters, it remains an open question whether the immune-modulating action of GH/IGF-I in fish indeed is restricted to the innate system or whether this current state of knowledge is due to an unbalanced data basis.

Thus far, only fragmentary data regarding the relevance of endocrine IGF-I (IGF-I serum levels) and/or paracrine/autocrine actions exists. The importance of systemic GH and IGF-I in the immune competence of fish, respectively, has been observed in the above mentioned channel catfish challenged with *Edwardsiella ictaluri* [28]. Here, lower IGF-I serum levels and down-regulated hepatic GHR expression were found. While treatment with recombinant bovine GH increased lysozyme activity, it did not elevate the IGF-I serum levels and GHR expression as compared to the control fish, which the authors attributed to the dietary regimen of this study. In addition, Bilodeau *et al.* [30] reported reduced mean plasma IGF-I levels in juvenile hybrid catfish after challenge with *Edwardsiella ictaluri*.

In light of the general importance of IGF-I during fish growth and organ development (e.g., [18,31]), not much is known on its role during development of the immune system. Thus, further studies should enlighten the potential interactions between GH/IGF and the immune systems, particularly during development.

First reports in human patients also point to a potential relevance of systemic IGF-I in immune competence: children who did not survive a septic shock possessed lower IGF-I serum levels than the survivors [32]. Furthermore, children during sepsis and septic shock also showed a suppression of IGF-I serum levels [33]. In both studies, high GH serum levels were measured, which were attributed to a stimulated GH release via the above mentioned feed-back mechanism or to increased endotoxin and cytokine load, respectively [32,33,34]. Furthermore, in HIV-infected patients, the IGF-I serum level gain was found to be a critical marker of the efficiency of GH treatment for the restoration of CD4+ T helper cells [35].

Thus, systemic elevation of GH and/or IGF-I levels seems to play a crucial role in the immune response of mammals and this may apply for bony fish as well as indicated from the few studies published so far.

However, local effects may exist that have not been sufficiently studied to date. Better knowledge in this field is necessary with respect to the increasing detection of the presence of hormones, their receptors and releasing hormones in immune cells themselves. This local presence of hormone systems within the immune system has recently been reviewed by Csaba [36], who highlighted the fact, that immune cells synthesize, store and secrete hormones identical to those of the endocrine glands, for example:

- pro-opio-melanocortin hormones;
- thyroid system hormones;
- GH and prolactin;
- melatonin, histamine, serotonin and catecholamines;
- gonadotropin-releasing hormones; and
- their respective receptors.

Both GH and prolactin, in addition to erythropoietin [37] and leptin, have been classified amongst the short-chain cytokines of the type 1 cytokine family, which supports the dual role of GH as a hormone and a cytokine (for further reading, see [38]).

In his review, Csaba [36] points out that the immune cells may be similar to the unicells (Tetrahymena), and thus retain the characteristics of this early phylogenetic level, while other cells during evolution accumulated together to form endocrine glands. As mobile cells, immune cells are able to transport the stored hormone to different locations (packed transport) or when attracted by local factors, accumulate in the region of the target, to synthesize and secrete hormones locally. The targeted packed transport is more economical than the hormone-release into the blood circulation by glandular endocrine organs.

However, while the distinct localization of IGF-I has been studied for many endocrine glands in mammals (e.g., [7,8,15]), as well as in fish (e.g., [13,14,15,16,31,39]), in the immune system, particularly in fish, it has not been systematically explored to date.

## 3. Immune Cells of Fish as Site of Local IGF Synthesis

Initial studies have detected IGF-I gene expression in immune organs of teleost fish and even found some correlation with infection or stress. However, the distinct localization at the cellular level has not been investigated in bony fish to date. Particularly since the head kidney is anatomically located nearby the excretory kidney, it has to be considered that it possesses a complex cellular composition. Different cell types express IGF-I as reported in tilapia, e.g., the cells of the *Oreochromis niloticus* renal tubules [31], or the *Oreochromis mossambicus* interrenal cells [40].

In addition, in the gilthead sea bream *Sparus aurata* and the rainbow trout *Oncorhynchus mykiss*, IGF-I gene was expressed in head kidney [41,42,43,44]. *In situ* hybridization of the tilapia head kidney revealed IGF-I mRNA in numerous immune cells [42,43,45]. In kidney and spleen of male and female yellow perch (*Perca flavescens*), a moderate expression of both IGF-I amplification products, IGF-Ia and IGF-Ib was detected [46]. The expression of both, IGF-I and IGF-II genes, has been described in adult tilapia spleen [42,43,45,47], gilthead sea bream and tilapia head kidney [41,42,43]. In red-banded sea bream head kidney and spleen, the expression levels were much lower for IGF-I than for IGF-II, which were similar to IGF-II expression levels in liver [48].

**Figure 1:**
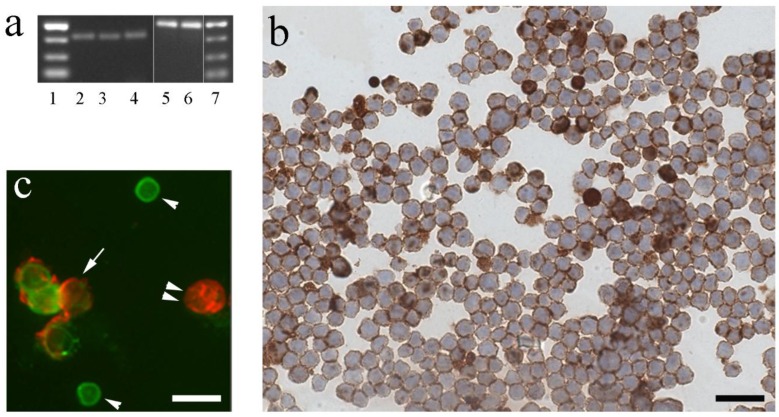
Detection of IGF-I in channel catfish (*I. punctatus)* and rainbow trout (*O. mykiss*) leukocytes. (**a**,**b**) IGF-I mRNA and peptide in *I. punctatus* T-cell line G14D using (**a**) PCR and (**b**) immunocytochemistry. (**a**) Agarose gels of real-time PCR products confirming the presence of IGF-I mRNA (bands 2–4) at the expected size (73 bp) and reference gene 18S rRNA (bands 5–6) at the expected size (85 bp). Bands 1 and 7: molecular weight marker. (**b**) The majority of the T-cells show IGF-I immunoreactive material (brown color) within the cytoplasm. Nuclei counterstained with haematoxylin. Bar: 60 µm. (**c**) Head kidney leukocytes freshly isolated from rainbow trout were adjusted to 1 × 10^7^ cells/mL, incubated with mouse mAb against rainbow trout granulocytes (mAbQ4E; [56]) visualized with a Texas red-coupled anti-mouse antibody as previously described [54]. Subsequently, cells were fixed for intracellular labeling for IGF-I using the same antiserum as in (**b**) followed by a FITC-coupled anti rabbit antibody for double immunofluorescence of IGF-I (green) in phagocytic cells (red, arrow). Note the small cells immunoreactive for IGF-I, which do not express the granulocyte marker (single arrowheads) and the large cell immunoreactive for the granulocyte marker, but without immunoreactivity for IGF-I (double arrow heads). Bar: 30 µm.

In order to further elucidate the distinct localization of IGF-I in leukocytes and to evaluate whether there exist analogies between higher and lower vertebrates, we investigated the presence of IGF-I in different leukocytes. For the detection of IGF-I mRNA (Figure 1a) and peptide (Figure 1b), we used T-cells from a defined Channel catfish (*Ictalurus punctatus*) T-cell line G14D established by Norman Miller, University of Mississippi Medical Center, Jackson, MS and co-workers [49]. Cells were purchased from ATCC (Cat.No: CRL-2759, [49]) and cultured according to protocol [50] in incomplete channel catfish medium supplemented with channel catfish serum kindly provided by Professor Miller. For real-time PCR (Figure 1a), primers and probes were created based on mRNA sequences of *I. punctatus* IGF-I (GenBank Access No: NM_001200295.1 [51]), and *O. mossambicus* 18S rRNA (GenBank Access No: AF497908.1) and obtained from Microsynth as described for other species and tissues (e.g., [42,43,47,52,53]). For immunocytochemistry (Figure 1b), cells from the same T-cell line G14D were fixed and by cytospin transferred to slides for immunostaining as described previously [54] using antiserum against IGF-I as described [31,55].

In order to investigate another fish species important in local aquaculture, we further detected IGF-I in head kidney leukocytes (Figure 1c) freshly isolated from adult female rainbow trout (*Oncorhynchus mykiss*) reared at the facilities of the Institute for Fish and Wildlife Health, University of Bern as described elsewhere [54]. In brief, the fish were kept under flow-through conditions (0.5 L/min) in air-saturated tap water (temperature 15 ± 1 °C). The head kidney leukocytes were characterized by an immunocytochemistry protocol [54] using a myeloid lineage marker specific monoclonal antibody [56], which recognizes a cell surface marker expressed on rainbow trout immune leukocytes ranging from immature myeloblasts to phagocyting cells, which are capable of respiratory burst. Thus, it can be considered as a common lineage marker for the myeloid lineage [56].

While IGF-I could be detected in T-cells from both fish species investigated, in contrast, in mature mammalian T-cells, intracellular IGF-I has not been detected to date (e.g., in the thymus, IGF-I was not found in T-cells and local IGF-I supply seems to be derived from thymus epithelial cells [57]). Nevertheless, its role in mammalian T-cell development appears to be pronounced (e.g., [58]). Most recently, in humans, we described the local presence of IGF-I in neutrophils of the tumor microenvironment of a lymphoma [59], which to the best of our knowledge was the first detection of IGF-I in granulocytes. Since we proposed an important role for IGF-I in cell shaping and restitution, e.g., of the high endothelial venules of human lymph nodes, we assume that IGF-I in phagocytic cells might be involved in cell growth, repair and proliferation, particularly of phagocytes. The production and/or local presence of IGF-I in macrophages have been previously demonstrated for humans and mice (e.g., [60,61]).

## 4. Regulation of IGF-I in Fish Immune Organs and Cells by GH and Other Physiological Factors

There is evidence also for regulation of extrahepatic IGF-I and IGF-II by GH [45,53,62,63,64,65,66,67] in numerous tissues, but minimal data exists about the immune organs. The GHR has been cloned and localized, for instance in the spleen of salmon [68], and rainbow trout [69], in head kidney and spleen and on hematopoetic cells of sea bream [41,70], and in tilapia spleen and head kidney [42]. GH-binding sites were detected by receptor autography in splenic melanomacrophage centers of gilthead sea bream [71] and in sea bass by immunohistochemistry in immune cells, which were morphologically identified from head kidney smears [21]. Here, aside from the different precursors of the erythropoietic line, also members of the myelocytic line exhibited GH binding capacity, particularly prominent in myeloblasts and to an appreciable extent in myelocytes. While mature eosinophils did not show binding of GH, immature eosinophils were labeled mainly at the periphery. A light binding was also observed in immature neutrophils while basophils were not detected on the smears which can be explained by their similar scarcity as in humans. Monocytes/macrophages which were very sparsely detectable, exhibited a faintly positive reaction for GH binding. Large and small lymphocytes also showed appreciable staining while no binding was detected in thrombocytes [21], which have been recently demonstrated to be capable of acting as phagocytic immune cells in teleost fish [72]. Interestingly, in rainbow trout, erythroid cells and also a subpopulation of B-lymphocytes responded to erythropoietin, while granulocytes and thrombocytes did not [37]. Thus, more studies are needed on stimuli of immune cell lineage differentiation and proliferation.

In rainbow trout spleen, the IGF-I steady-state levels were unaffected by GH treatment and only increased over time [66]. In the tilapia *O. niloticus*, GH injections increased IGF-I mRNA in the head kidney, even more than in the liver, which is the main source of endocrine IGF-I. This elevation in the head kidney could be morphologically verified by *in situ* hybridization. Here again, IGF-I mRNA could be localized in numerous immune cells. IGF-II and GHR gene expressions were not affected by GH treatment [43]. In the spleen, also no change occurred in GHR mRNA, but both, IGF-I and IGF-II mRNAs were increased as could be localized by *in situ* hybridization where IGF-I mRNA was increased in immune cells.

However, the immunological potential of local IGF-I and potential stimulation by GH treatment remains to be investigated. Thus far, in the head kidney of *Enteromyxum leei*-infected gilthead sea bream (*Sparus aurata*), IGF-I gene expression declined, which was accompanied by a down-regulation also of the GHR expression. In contrast, the non-infected individuals showed increased IGF-I and GHR expression levels. From these results, the authors concluded that an increase in IGF-I and GHR expression might be advantageous for immune cells in response to the parasite [41].

The role of autocrine/paracrine IGF-I as related to endocrine IGF-I is also not that clear to date [53,73], and even less is known with respect to fish health. Disturbances of fish health, e.g., by stress and infection, might alter the GH/IGF-I axis at different levels and, thus, impact fish growth, however potential interactions among stress, health and growth are generally extremely complex and not much is known thus far as summarized by Beckman [73]. Thus far, in sea bream during vibriosis infection, the local IGF-I gene expression in liver and kidney was down-regulated [74], but whether this may be due to the general stress situation of the organism or to alterations of the GH/IGF-I axis or specifically induced by the infection remains to be clarified. For instance, also in gilthead sea bream exposed to confinement stress, IGF-I gene expression was declined in the liver [75] and rearing and handling conditions as well as many other factors impact the complex fish physiology, in particular the GH/IGF-I axis [73].

A potential crosstalk between IGF-I and other growth factors and cytokines is coming into focus for research in mammals (e.g., [76]), and this might be of particular interest also for fish research.

As mentioned above, human and murine myeloid cells, particularly macrophages, produce IGF-I in relatively large amounts [60,61], also stimulated by cytokines such as tumor necrosis factor (TNF)-α [77,78], which was also reported to induce a state of resistance against IGF-I [79]. *Vice versa*, there exist indications that IGF-I counteracted sickness behavior and depression in mice induced by cytokines including TNF-α [80]. However, data are rudimentary and even contradictory: in GH-treated growth-deficient children altered metabolic parameters including TNF-α levels are reported e.g., [81], and modulation of cytokines by GH at high doses is suggested to be involved in adverse consequences in critically ill patients, such as septic shock and uncontrolled infections [82].

Thus, the idea of a bidirectional communication between neuroendocrine and immune system exists and demands a search for better knowledge also in fish [38]. While different cytokines have already been explored in fish (for reviews, see [83,84]), data on potential interactions with the GH/IGF-I axis are still very fragmentary and mainly deal with TNF-α: e.g., in GH-injected tilapia, TNF-α expression was elevated in the liver [43] and in a GH-RH-treated flounder embryonic cell line, TNF-α mRNA was also induced, while in a primary pituitary cell culture, TNF-α mRNA was decreased [85].

## 5. IGF-I and TNF-α during Pathogenic Infection of Fish

A possible role of IGF-I and TNF-α in infection and the capability of the host to cope with tissue damage has been previously demonstrated by treating developing rainbow trout, prior to infection with *Yersinia ruckeri*, with 17β-estradiol (E2). The effects of either E2-treatment or the bacterial infection or combination of both on the expression pattern of cytokines (*i.e.*, TNF-α) and/or growth factors (*i.e.*, IGF-I) was tissue and treatment specific [44]. The results indicate a stimulatory effect of infection on IGF-I and TNF-α gene expression in the spleen: IGF-I was elevated 6.802-fold (*p* = 0.0001) at Day 3 after infection with 10^6^ colony-forming unit (CFU) of *Yersinia ruckeri* [86,87] and TNF-α gene expression 60.497-fold (*p* = 0.001) as compared with the uninfected control [44]. The infection reduced the survival rates of the rainbow trout to 61% [87]. In contrast, in the head kidney, three days after infection both, IGF-I and TNF-α were lowered, IGF-I to 0.093-fold (*p* = 0.0001), and TNF-α to 0.079-fold (*p* = 0.0001), respectively. IGF-I and TNF-α gene expressions were lower also prior to infection already after the developmental E2-treatment. Both treatments induced additive effects [44].

There exists evidence for interactions between the reproductive state and fish health, particularly the immune system (reviewed by [5,14,38,88]). The suppression of IGF-I by E2 in head kidney was consistent with previous findings by our group where IGF-I was still lower even 1 month after termination of the estrogen treatment [42]. Interestingly, both IGF-I and TNF-α reacted in parallel to both stimuli, E2 treatment and infection [44]. In addition, after E2-treatment of juvenile sea bass (*Dicentrarchus labrax*), suppressed TNF-α gene expression in head kidney leukocytes was reported [89].

In the spleen, we observed a stimulatory effect of infection with Yersinia on both, IGF-I and TNF-α gene expressions with a dose-dependent increase in the E2-treated groups. Particularly TNF-α expression levels were massively elevated by the infection (60.497-fold in the group without E2 pre-treatment, 322.568-fold in the low E2 pre-treatment, 5651.008-fold in the high E2 pre-treatment group) [44]. However, the lack of response of IGF-I gene expression to estrogen treatment was different from previous findings by our group where IGF-I was strongly suppressed even one month after cessation of treatment [42].

In conclusion, the results of the study point to tissue specific effects of either treatment. While in liver and head kidney, the effects were suppressive on both, IGF-I and TNF-α expression levels, in spleen, the gene expression levels were stimulated. Thus, the proposed high TNF-α expression as an indicator for the degree of infection in rainbow trout in another study [90] may be counteracted by the spleen with a parallel elevation of IGF-I, although to a lesser extent. This local elevation of IGF-I as described [44] may be supportive for survival in fish as previously suggested by other studies in humans and fish. Further studies are needed to explore the complex interactions between cytokines and growth factors (for reviews on research in fish see, e.g., [5,6,10,12,38,83,91]).

## 6. Conclusions

Numerous indications derived from studies in different fish species point to interactions between the GH/IGF and the immune system. Although most data support the current assumption of a general stimulatory action of GH and IGF-I on immune cell expansion and functional parameters, it remains an open question whether the immune-modulating action of GH/IGF-I in fish indeed is restricted to the innate system or whether this is due to the thus far available data basis. Our detection of IGF-I in channel catfish T-lymphocytes points to a role also in the adaptive immune system.

More studies are required to take into consideration different life traits, environments, and pathogens in order to better compare species differences. In particular, studies are needed to investigate why positive influences of GH on different immune functions do not generally lead to an efficient immune response and an improved health status. A better understanding of this phenomenon may be revealed by research into the role of the GH/IGF-I axis during development of fish immune organs and cell subtypes. Here too, more tools to characterize cell lineages and the increasing number of cytokines detected in bony fish will also contribute to a better understanding of the complex orchestration of cytokines and growth factors in the fish immune system.
